# A combination of Dihydroartemisinin and Venetoclax enhances antitumor effect in AML via C-MYC/BCL-XL/MCL-1 triple targeting

**DOI:** 10.1007/s12672-025-02242-7

**Published:** 2025-04-09

**Authors:** Fenglin Li, Yao Chen, Haihui Zhuang, Renzhi Pei, Yuyu Lu, Dong chen, Shuangyue Li, Peipei Ye, Jiaying lian, Ying Lu

**Affiliations:** 1 Department of Hematology, The Affiliated People’s Hospital of Ningbo University, Baizhang Road 251#, Ningbo, China; 2 Shaoxing Central Hospital, Shaoxing, China; 3 Institute of Hematology, Ningbo University, Ningbo, China

**Keywords:** AML VEN, DHA, MCL-1, C-MYC

## Abstract

**Background:**

Acute myeloid leukemia (AML) is associated with high rates of resistance to standard therapies, necessitating the exploration of novel treatment strategies. Venetoclax (VEN) has shown efficacy in AML, yet drug resistance remains a significant challenge. This study aims to explore the synergistic effects of combining dihydroartemisinin (DHA) with VEN to improve therapeutic outcomes in AML.

**Methods:**

AML cell lines and primary cells from AML patients were treated with various concentrations of DHA, VEN and their combined regimen. The cytotoxic effects were evaluated using MTS assays, flow cytometry for apoptosis analysis, and cell cycle assessments. Protein levels of caspase-3, PARP, MCL-1, BCL-XL and C-MYC were analyzed to elucidate the underlying mechanisms of the observed synergy.

**Results:**

The combination of VEN and DHA demonstrated a significant synergistic cytotoxic effect on AML cells, characterized by reduced cell proliferation, induced apoptosis, and cell cycle arrest in the G0/G1 phase. Mechanistically, the synergy was associated with increased levels of cleaved caspase-3 and PARP, along with the downregulation of anti-apoptotic proteins MCL-1 and BCL-XL. Additionally, the combined treatment led to a significant decrease in C-MYC expression. This synergistic effect was consistently observed across all primary AML patient samples analyzed.

**Conclusion:**

The findings suggest that the combination of VEN and DHA exerts synergistic anti-leukemic effects by targeting BCL-XL, MCL-1 and C-MYC, offering a promising therapeutic strategy for AML.

**Supplementary Information:**

The online version contains supplementary material available at 10.1007/s12672-025-02242-7.

## Introduction

Acute Myeloid Leukemia (AML) is a hematologic malignancy characterized by clonal proliferation and impaired differentiation of myeloid hematopoietic cells [1, 2]. The prognosis for AML patients is poor, with a five-year survival rate of just 24% and a median overall survival time of only 8.5 months [3]. The standard treatment for younger patients is the "3 + 7" regimen, which consists of three days of anthracycline-based drugs followed by seven days of cytarabine [4, 5]. However, older patients often struggle to tolerate intensive chemotherapy, which leaves them with limited treatment options and poorer outcomes [6, 7]. Consequently, there is an urgent need to explore novel and effective treatment strategies to improve survival rates in elderly AML patients.

Venetoclax (VEN) is a promising new oral medication that selectively inhibits B-cell lymphoma-2 (BCL-2), thereby triggering apoptosis in cancer cells through the activation of the intrinsic mitochondrial apoptotic pathway [8]. BCL-2 is often overexpressed in various types of tumors, particularly in hematologic malignancies like AML, chronic lymphocytic leukemia, and follicular lymphoma [9]. This overexpression is strongly linked to tumor development, progression, and resistance to chemotherapy. VEN received accelerated approval in 2018 and regular approval in 2020 [10, 11]. However, a significant proportion of patients develop primary or acquired resistance to VEN [12]. A key factor contributing to this resistance is the compensatory increase in MCL-1 expression due to continuous inhibition of BCL-2 [13, 14]. Hence, the inhibition or downregulation of MCL-1 protein is an important strategy to enhance the efficacy of VEN.

Due to their low toxicity and multi-target properties, there has been increasing interest in exploring the anti-tumor activities of natural compounds. Dihydroartemisinin (DHA) is extracted from Artemisia annua (sweet wormwood). It exhibits higher pharmacological activity and low toxicity compared to traditional artemisinin [15–17]. In AML cells, DHA has been shown to induce apoptosis and inhibit proliferation by suppressing the PTEN/AKT signaling pathway [18, 19]. Additionally, Nazmabadi et al. found that DHA can increase the sensitivity of U937 and KG-1 cells to VEN. However, this study did not validate the results on primary AML samples and only detected the mRNA expression of BCL-2 and MCL-1 using RT-qPCR [20].

This study aims to further confirm the synergistic effects of combining VEN and DHA in other AML cell lines and primary AML samples. First, we assessed whether different concentrations of the two drugs produced synergistic effects on MOLM-13 and MV4-11 cells, as well as specifically on primary AML samples. Subsequently, the impact of the combination on cell proliferation, apoptosis, and the cell cycle was evaluated. The mechanisms underlying the observed synergy were also explored using Western blot analysis. Our results indicate that the combination of VEN and DHA produces significant synergistic cytotoxicity in AML cell lines and primary AML cells, effectively inhibiting cell proliferation, enhancing apoptosis, and inducing cell cycle arrest in the G0/G1 phase. The underlying mechanism for this synergistic effect is attributed to the downregulation of the anti-apoptotic proteins BCL-XL, MCL-1, and C-MYC, thus increasing the vulnerability of AML cells to VEN and promoting apoptotic processes.

Methods

### Cell lines and primary cells

The AML cell lines MV4-11, MOLM-13 and THP-1 were generously provided by Jie Jin from The First Affiliated Hospital of Zhejiang University School of Medicine in Hangzhou, China. All cells were cultured in Iscove’s Modified Dulbecco’s Medium (IMDM) (Gibco, USA), supplemented with 20% Fetal Bovine Serum (FBS) (Gibco, USA), and an antibiotic solution containing 100 U/mL penicillin and 100 µg/mL streptomycin. Cells were cultured in a 37 °C incubator with 5% CO₂, and routine splitting was performed every three days to ensure they remained in the exponential growth phase.

Bone marrow or peripheral blood samples were collected from patients diagnosed with de novo AML at the Affiliated People’s Hospital of Ningbo University. Written informed consent was obtained from each patient following the Declaration of Helsinki, and the study received approval from the Clinical Research Ethics Committee of the hospital. Mononuclear cells were isolated from eight AML patients using Ficoll-Hypaque density gradient centrifugation and cultured in IMDM supplemented with 10% FBS [21]. Detailed information regarding the patient characteristics is provided in Table S1.

### Agents and antibodies

VEN and DHA were purchased from Selleckchem (Houston, TX, USA) and dissolved in DMSO for in vitro assays. The antibodies BCL-XL (#2764), MCL-1 (#39224), BCL-2 (#4223), C-MYC/N-MYC (#13987), PARP (#9532), caspase-3 (#9662), and GAPDH (#5174) were obtained from Cell Signaling Technology (Beverly, MA, USA). The secondary antibodies, Goat Anti-Rabbit (P21118) and Goat Anti-Mouse (Q20329) were purchased from TransGen Biotech (Beijing, China).

### Cell viability

For the cell viability assessment, 15,000 AML cells (cell density of 1.5 × 10^5^ cells/mL) or 100,000 primary AML cells (cell density of 1.0 × 10^6^ cells/mL) were seeded in a 96-well plate and treated with designated drugs for either 24 or 48 h. Subsequently, 20 µL of MTS (Promega Corporation, USA) solution was added to each well and incubated for an additional four hours at 37 °C. The absorbance was measured at 490 nm. The half-maximal inhibitory concentration (IC50) values and combination indices (CI) were determined using CalcuSyn Software. The CI was utilized to analyze the effects of drug combinations. A CI value less than 1 indicates synergy; a CI value of 1 suggests an additive effect; and a CI value greater than 1 denotes antagonism. ED (Dose effect)50: The CI value for the combination of two drugs that results in 50% cell death. ED75: The CI value for the combination of two drugs that results in 75% cell death. ED90: The CI value for the combination of two drugs that results in 90% cell death.

### Flow cytometry

Apoptosis was quantified using an apoptosis detection kit (MultiSciences Biotech, Hangzhou, China) according to the manufacturer’s protocol. For MV4-11, the concentrations were set at 50 nM for VEN and 625 nM for DHA, with the combination group treated with both agents. For MOLM-13, the concentrations were 80 nM for VEN and 800 nM for DHA, also treated in combination. After treated for 24 h or 48 h, cells were harvested and washed with PBS. The cells were resuspended in a binding buffer and labeled with Annexin V-FITC and propidium iodide (PI) in the dark at room temperature for 15 min. Subsequently, samples were analyzed using a FACScan^™^ flow cytometer (Becton Dickinson, San Diego, CA, USA), with results presented as the percentage of Annexin V positive cells [22].

### Western blot

After treated with 50 nM VEN, 625 nM DHA, or a combination for 48 h, cells were harvested, washed with PBS, and lysed in RIPA (Solarbio, Beijing, China) buffer containing protease and phosphatase inhibitors on ice for 30 min. The protein concentration was measured using a Bicinchoninic Acid kit (BCA, Beyotime, Shanghai, China). Equal amounts of protein were resolved on 8–12% sodium dodecyl sulfate (SDS)-polyacrylamide gels and transferred to polyvinylidene difluoride (PVDF) membranes. The membranes were blocked with 5% non-fat milk in TBS-T for one hour and incubated with primary antibodies overnight at 4 °C. After thorough washing with TBS-T, the membranes were incubated with the appropriate secondary antibodies for two hours at room temperature. Protein visualization was achieved using an ECL detection kit (NCM Biotech, Suzhou, China), followed by analysis with a ChemiScope 6100 imaging system (Clinx, Shanghai, China) [23]. Densitometry measurements were carried out using Image J software. GAPDH was utilized as a loading control. Subsequently, data were normalized by setting the control group to 1. Representative blots from three independent experiments, along with their corresponding densitometric, analyses are presented as means ± standard deviation.

### Statistical analysis

Statistical significance was assessed using one-way ANOVA. Results are expressed as means ± standard deviation (SD) from three independent experiments. Statistical analyses were conducted using Prism software v9.5 (GraphPad Software, La Jolla, CA, USA) and IBM SPSS Statistics 22 (Armonk, NY, USA). P < 0.05 was considered statistically significant (P < 0.05*, P < 0.01**, P < 0.001***).

## Results

### Synergistic anti-leukemic effects of Dihydroartemisinin and Venetoclax in AML cells

**Fig. 1:**
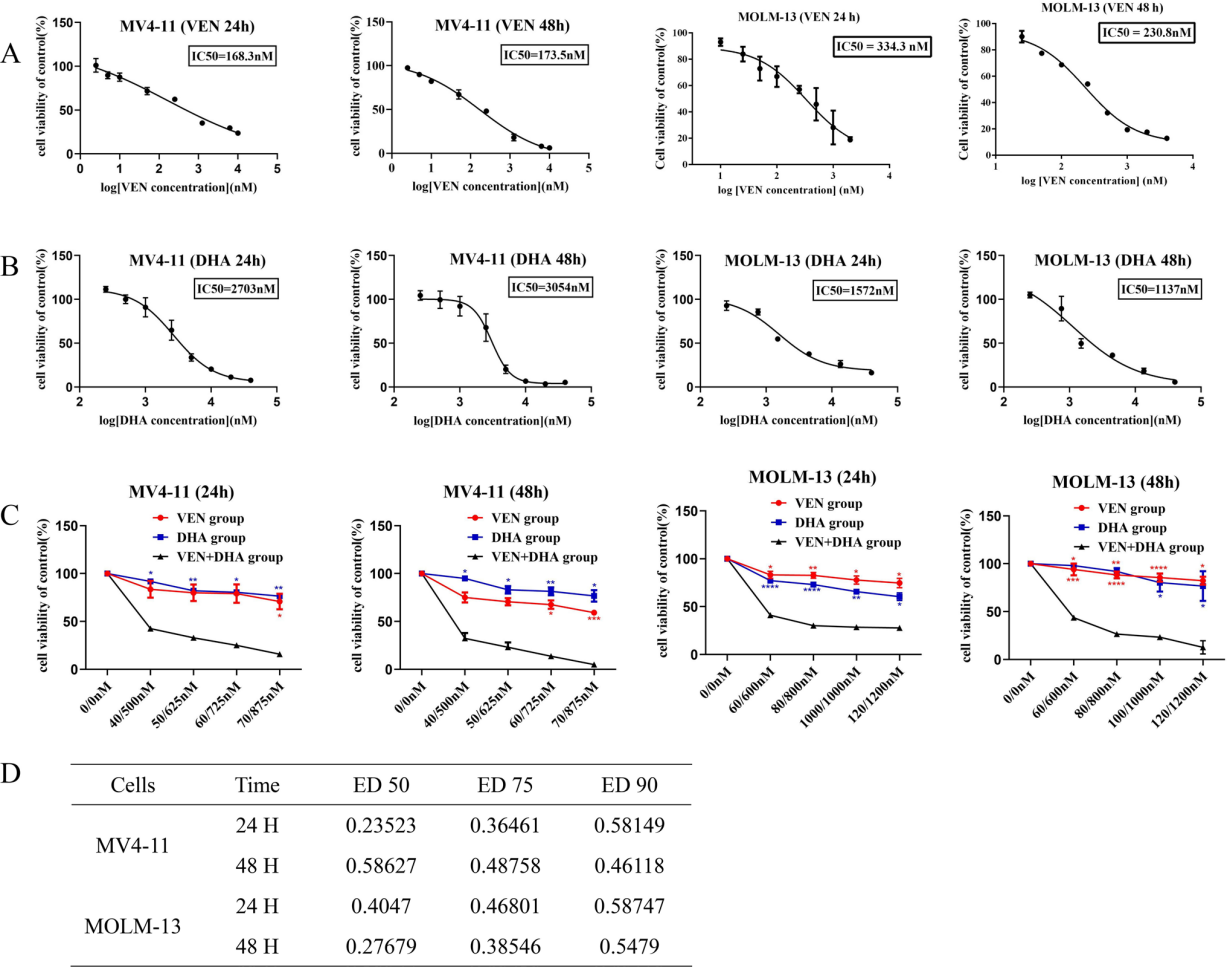
Synergistic Anti-Leukemic Effects of Dihydroartemisinin and Venetoclax in AML Cells. **A** The IC50 of VEN on MV4-11 and MOLM-13 cell lines was measured by MTS after 24 and 48 h of treatment. **B** The IC50 of DHA on MOLM-13 and MV4-11 cell lines was measured by MTS after 24 and 48 h of treatment. **C** Cell viability assessment of MV4-11 and MOLM-13 cells treated with VEN or DHA alone or in combination for 24 or 48 h. The concentrations of VEN in the MV4-11 cell line were 40 nM, 50 nM, 60 nM, and 70 nM. The concentrations of DHA in the MV4-11 cell line were 500 nM, 625 nM, 725 nM, and 875 nM. The concentrations of VEN in the MOLM-13 cell line were 60 nM, 80 nM, 100 nM, and 120 nM. The concentrations of DHA in the MOLM-13 cell line were 600 nM, 800 nM, 1000 nM, and 1200 nM. **D** The combination index (CI) for the combination of VEN and DHA on MV4-11 and MOLM-13 cells at 24 and 48 h. P < 0.05 was considered statistically significant (P < 0.05*, P < 0.01**, P < 0.001***)

To evaluate the sensitivity of AML cells to VEN and DHA, MV4-11, MOLM-13 and THP-1 cell lines were treated with VEN and DHA for 24 or 48 h. It was revealed that increasing concentrations of VEN or DHA resulted in a gradual decline in cell viability, demonstrating their cytotoxic effects on both MV4-11 and MOLM-13 cells in a dose-dependent manner. The half-maximal inhibitory concentrations (IC50) for these cell lines were calculated (Fig. 1A-B).

Subsequently, the synergistic anti-leukemic effect of the combination treatment with VEN and DHA was assessed. AML cells were exposed to various concentrations of VEN, DHA, and their combination for 24 or 48 h. The results indicated that significantly stronger cytotoxic effects were exhibited by the combination treatment on MV4-11 and MOLM-13 cells compared to individual drug treatments, with a combination index (CI) of less than 1 (Fig. 1C-D). Furthermore, the synergistic effect was also observed in the THP-1 cell line, which was relatively insensitive to VEN (Fig. S1). This suggested that a synergistic cytotoxic effect on AML cell lines was induced by the combination of VEN and DHA.

### Synergistic anti-leukemic effect in primary AML samples

**Fig. 2:**
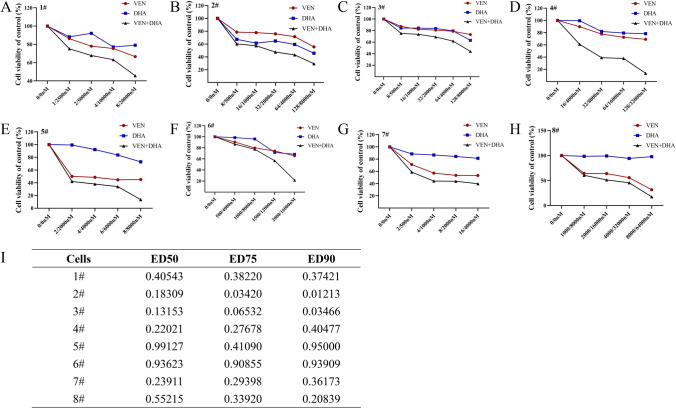
Anti-Leukemic Effect in Primary AML Samples. **A**–H Cell viability assessment of 8 primary AML samples treated with VEN or DHA alone or in combination for 24 h. The x-axis represents the combined concentration of the two drugs, with the concentration of VEN displayed on the left and the concentration of DHA on the right. **I** The CI for the interaction of VEN and DHA on 8 primary AML samples at 24 h

The synergistic effects of the VEN and DHA combination were further validated in primary AML patient samples. It was indicated that both VEN and DHA effectively inhibited the viability of primary AML cells, with synergy (CI < 1.0) observed across eight primary AML samples (Fig. 2). Detailed patient demographics were provided in the supplementary materials.

### Inhibition of cell proliferation in AML Cells

**Fig. 3:**
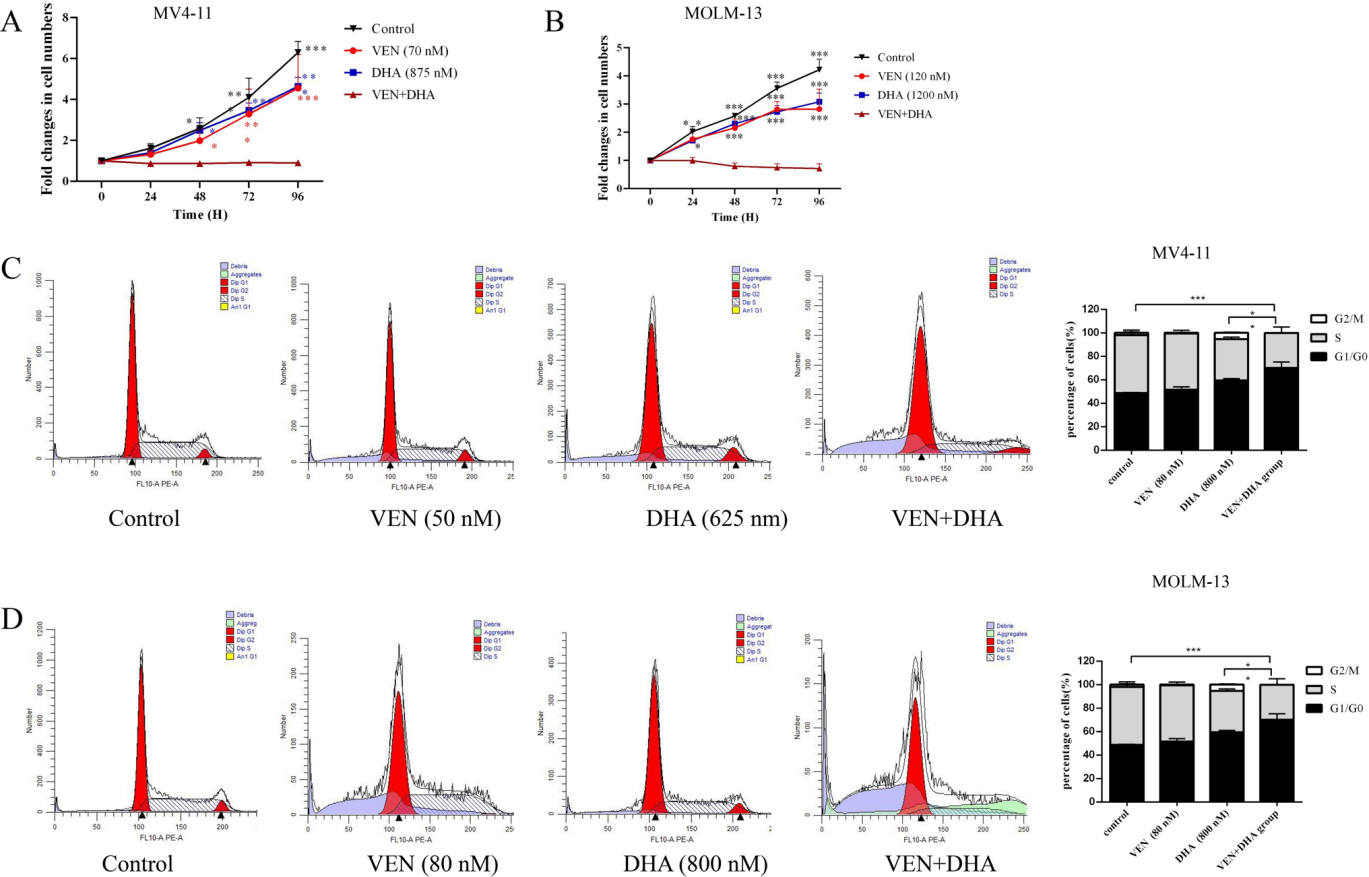
Inhibition of Cell Proliferation in AML Cells. **A**-**B** The growth of MV4-11 and MOLM-13 cells was significantly inhibited following treatment with the combination of VEN and DHA. **C**-**D** Flow cytometry analysis revealed a notable arrest of MV4-11 and MOLM-13 cells in the G1/G0 phase after 24 h of combination treatment. Results are expressed as means ± standard deviation (SD) from three independent experiments. P < 0.05 was considered statistically significant (P < 0.05*, P < 0.01**, P < 0.001***)

Given the synergistic cytotoxic effect of VEN and DHA on AML cells, analysis of cell proliferation and the cell cycle was performed. Both the VEN and DHA monotreatment groups exhibited partial inhibition of cell proliferation, while the combination treatment group nearly completely halted the growth of MV4-11 and MOLM-13 cells (Fig. 3A-B). The results from cell cycle analysis indicated that VEN alone did not have a significant effect on cell cycle arrest, whereas DHA caused AML cells to be arrested in the G1/G0 phase. The combination treatment group showed a significantly higher proportion of cells in the G1/G0 phase compared to the DHA monotreatment group in MV4-11cells (Fig. 3C) and MOLM-13 cells (Fig. 3D). These results demonstrated that the combination of VEN and DHA effectively inhibited cell proliferation in AML cells.

### Induction of apoptosis in AML Cells

**Fig. 4:**
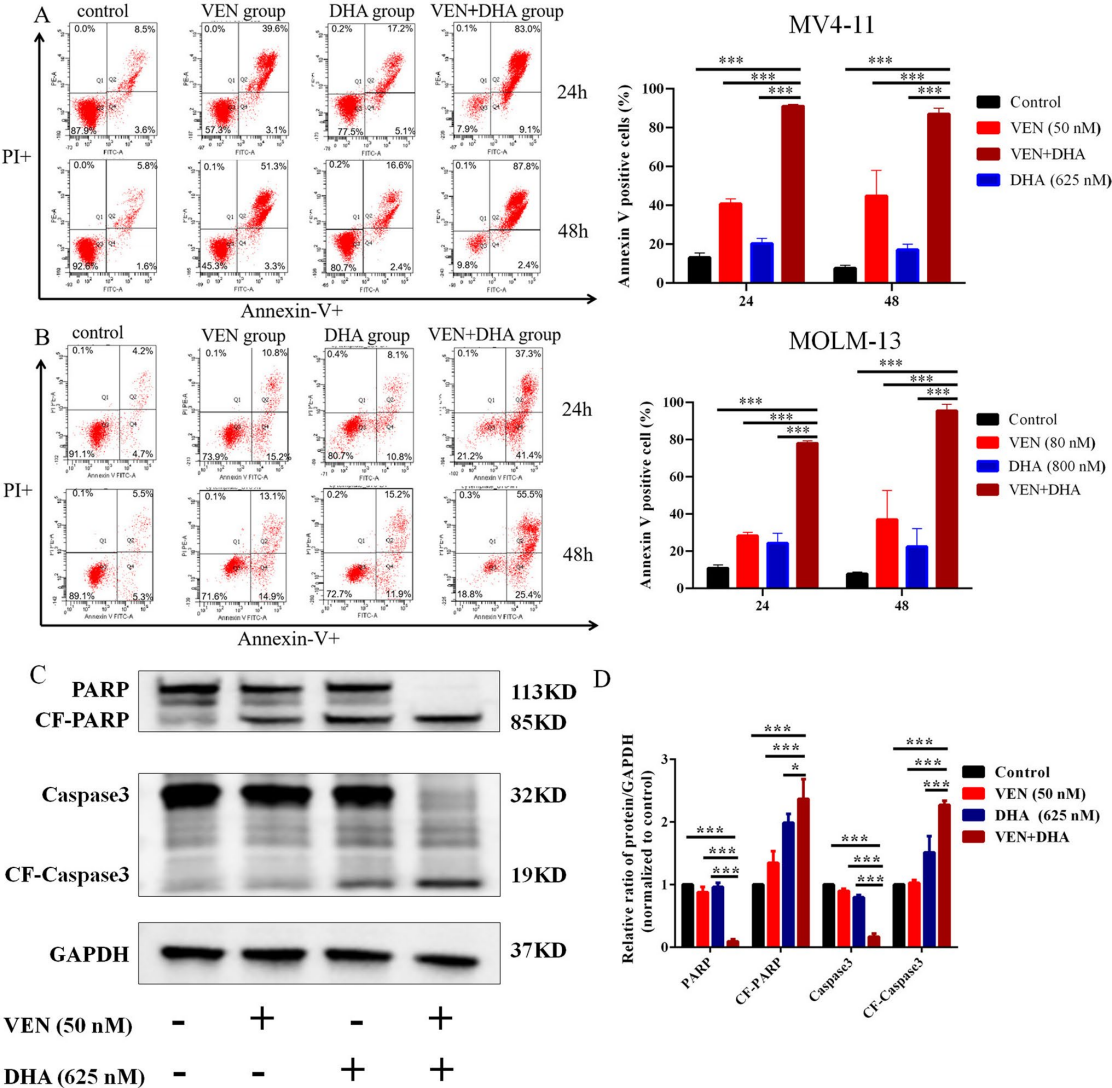
Induction of Apoptosis in AML Cells. **A**-**B** The rates of apoptosis in MV4-11 and MOLM-13 cells significantly increased following treatment with the combined VEN and DHA treatment. **C** Western blot analysis showed significant cleavage of apoptosis-related proteins PARP and Caspase-3 in cells treated with the combination treatment after 24 h. **D** GAPDH was utilized as a loading control. Subsequently data were normalized by setting the control group to 1. Representative blots from three independent experiments, along with their corresponding densitometric analyses, are presented as means ± standard deviation. P < 0.05 was considered statistically significant (P < 0.05*, P < 0.01**, P < 0.001***)

Considering that VEN acts as a BCL-2 inhibitor and exerts anti-leukemic effects through apoptosis, the impact of the combination treatment on apoptosis in AML cells was investigated. MV4-11 and MOLM-13 cells were treated with VEN and DHA, either alone or in combination, for 24 or 48 h. It was shown that the combination treatment significantly increased the apoptotic rate in both cell lines compared to the monotreatment and control groups (P < 0.0001) (Fig. 4A-B). Moreover, western blot analysis revealed that levels of cleaved PARP and cleaved caspase-3 were notably higher in the combination treatment group than in the monotherapy groups (Fig. 4C-D), indicating that the combination more effectively induced apoptosis in AML cells.

### Mechanism of synergistic effects by inhibiting C-MYC, BCL-XL, and MCL-1 proteins

**Fig. 5:**
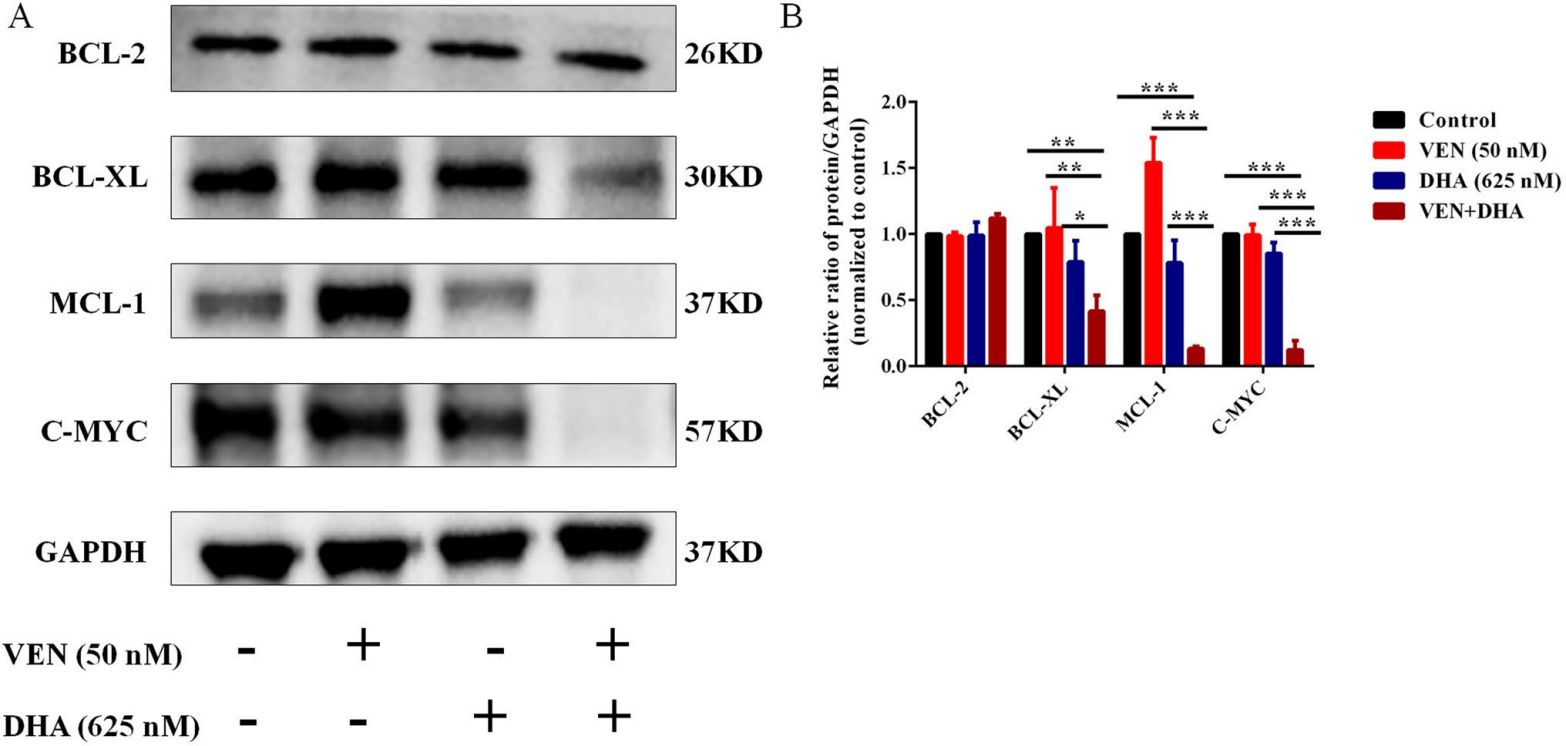
Mechanism of Synergistic Effects by Inhibiting C-MYC, BCL-XL and MCL-1 Proteins. **A** Western blot analysis showed that MCL-1 was upregulated in the VEN monotreatment group, downregulated in the DHA monotreatment group, and further downregulated in the combined treatment group. Additionally, BCL-XL and C-MYC protein levels were also decreased in the combination group. **B** GAPDH was utilized as a loading control. Subsequently, data were normalized by setting the control group to 1. Representative blots from three independent experiments, along with their corresponding densitometric analyses, are presented as means ± standard deviation. P < 0.05 was considered statistically significant (P < 0.05*, P < 0.01**, P < 0.001***)

As MCL-1 is a vital player in resistance to VEN in AML cells, the expression of the MCL-1 protein was examined in this study. The results indicated that treatment with VEN led to an upregulation of MCL-1, while DHA partially downregulated this protein. Notably, MCL-1 expression was significantly reduced in the combination group. Additionally, levels of BCL-XL and C-MYC were also significantly downregulated (Fig. 5A-B). These findings suggested that the combination of VEN and DHA achieved a synergistic cytotoxic effect on AML cells by decreasing the levels of MCL-1, BCL-XL and C-MYC.

## Discussion

In this study, we aimed to investigate the potential synergistic effects of VEN and DHA in the treatment of AML, which could provide one strategy to overcome VEN resistance. One of the main mechanisms of VEN resistance was identified as the compensatory upregulation of the anti-apoptotic protein MCL-1. Previous research had established that Dihydroartemisinin (DHA) could directly downregulate MCL-1 expression. Given this insight, the potential synergistic effects of combining DHA with VEN in AML treatment were explored. The findings indicated that the combination of these drugs significantly inhibited AML cell proliferation, induced cell cycle arrest at the G1/G0 phase, and promoted apoptosis. Importantly, the combination treatment led to a marked reduction in the expression levels of MCL-1, BCL-XL and C-MYC.

DHA, a compound derived from traditional Chinese medicine, has demonstrated significant cytotoxic effects on AML cells. It effectively inhibited cell proliferation, induced cell cycle arrest in the G1/G0 phases, and promoted ferroptosis in AML cells [24]. Research by Gao et al. indicated that DHA could enhance cell apoptosis and decrease MCL-1 protein levels in a concentration- and time-dependent manner, primarily by inhibiting the MAPKK/ERK signaling pathway [18]. Similarly, Yan et al. showed that DHA suppressed MCL-1 expression in non-small cell lung cancer through the JAK/STAT3 pathway [25]. The findings of this study were consistent with these previous studies, confirming that DHA induced apoptosis in AML cells while reducing MCL-1 protein levels. The apoptotic effects of DHA were mediated through the activation of the Caspase-3/PARP pathway. Together, these observations highlighted DHA's significant role in promoting apoptosis and downregulating MCL-1 in AML cells.

The challenge of treating AML with VEN was exacerbated by various resistance mechanisms [13, 14]. Studies indicated that treating cell lines with escalating doses of VEN led to increased expression levels of MCL-1 and BCL-XL [26, 27]. Nazmabadi et al. demonstrated that DHA enhanced the sensitivity of U937 and KG-1 cells to VEN, although this study did not validate its findings using primary AML samples [20]. Our research expanded on previous work by confirming the significant synergistic effects of combining VEN and DHA in other AML cell lines and primary AML patient samples. While Roya's study indicated that DHA could reduce the mRNA levels of BCL-2 and MCL-1, our findings revealed that the combination of VEN and DHA significantly decreased the protein levels of both MCL-1 and BCL-XL. Studies by Wang et al. further support our findings, showing that the combination of MCL-1 or BCL-XL inhibitors with VEN enhanced therapeutic responses in AML cell lines [28]. These findings highlight that the synergy between VEN and DHA is likely mediated through the downregulation of MCL-1 and BCL-XL, suggesting that this combination may improve sensitivity to VEN.

Moreover, a significant reduction in C-MYC protein levels was observed following the combined treatment. C-MYC is a transcription factor that regulates numerous biological processes, including cell proliferation and apoptosis [29]. Studies indicated that knocking out C-MYC promoted myeloid differentiation and inhibited AML cell proliferation, while high C-MYC expression was associated with the development and resistance of AML [30, 31]. Targeting C-MYC has emerged as a promising therapeutic approach in AML. Previous studies reported that the combination of the MCL-1 selective inhibitor AZD5991 and the C-MYC inhibitor (10058-F4 or S63845) showed promising and synergistic antileukemic activity in AML cell lines that had acquired resistance to Cytarabine(Ara-C) and in primary AML cells derived from a patient who relapsed post-chemotherapy [32, 33]. Thus, the observed downregulation of both C-MYC and MCL-1 following the combination of VEN and DHA suggested a multifaceted mechanism driving their synergistic effects, pointing to a potential for enhanced therapeutic efficacy in AML treatment. Further investigations into the interactions between these proteins were deemed crucial for refining strategies to overcome resistance.

## Conclusion

This study demonstrated that the combination of VEN and DHA exerted a synergistic cytotoxic effect on AML. This combined treatment effectively inhibited essential behaviors of AML cells, including cell proliferation, induction of apoptosis, and arrest of the cell cycle in the G0/G1 phase. The underlying mechanism for this synergy appeared to involve the downregulation of the anti-apoptotic proteins MCL-1 and BCL-XL. Importantly, a noteworthy reduction in the expression levels of the oncogene C-MYC was also observed following the combined treatment. This synergistic effect was consistently noted across all eight primary AML patient samples that were analyzed, indicating the broad potential of this approach. However, certain limitations were present in our study, as in vivo experiments to evaluate the effects of this drug combination were not conducted.

## Supplementary Information

Additional file 1.

Additional file 2.

## Data Availability

All data supporting the findings of this study are available within the paper and its Supplementary Information.
