# Supplementary material for: A combination of Dihydroartemisinin and Venetoclax enhances antitumor effect in AML via C-MYC/BCL-XL/MCL-1 triple targeting
Source: Discov Oncol. 2025 Apr 9;16:496. doi: 10.1007/s12672-025-02242-7 (PMC11982003; doi:10.1007/s12672-025-02242-7)
Supplement: Supplementary file 1 — Additional file 1. [file 12672_2025_2242_MOESM1_ESM.docx]

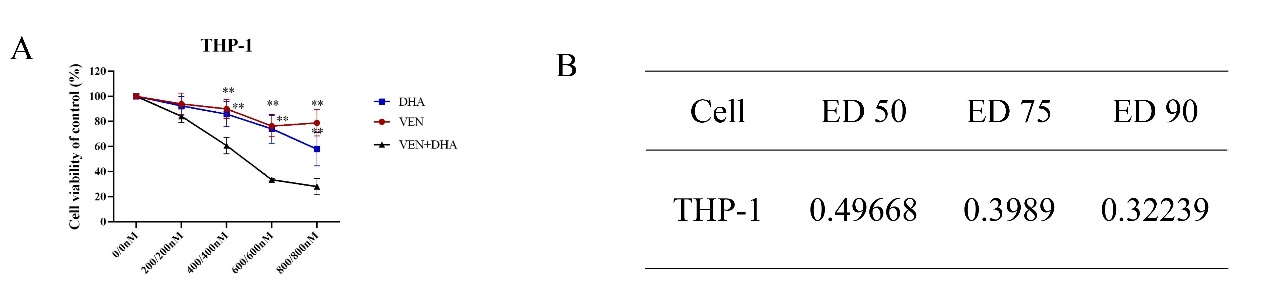


Fig S1. Synergistic Anti-Leukemic Effects of Dihydroartemisinin and Venetoclax in THP-1 Cells

A. Cell viability assessment of THP-1 cells treated with VEN or DHA alone or in combination for 48 hours. The drug concentrations of VEN and DHA in the THP-1 cell line were 200, 400, 600, and 800 nM.

B. D. The combination index (CI) for the interaction of VEN and DHA on THP-1 cells at 48 hours. P < 0.05 was considered statistically significant (P < 0.05 *, P < 0.01 **, P < 0.001 ***).
