# Supplementary material for: A combination of Dihydroartemisinin and Venetoclax enhances antitumor effect in AML via C-MYC/BCL-XL/MCL-1 triple targeting
Source: Discov Oncol. 2025 Apr 9;16:496. doi: 10.1007/s12672-025-02242-7 (PMC11982003; doi:10.1007/s12672-025-02242-7)
Supplement: Supplementary file 2 — Additional file 2. [file 12672_2025_2242_MOESM2_ESM.docx]

| Number | Sex | Age | FAB Type | chromosome | gene expression or mutations | Therapy program^1^ | CR | Outcome |
| --- | --- | --- | --- | --- | --- | --- | --- | --- |
| Patient.1# | Male | 54 | M5 | 46，XY | FLT3-ITD(+)，MLL-PTD(+)，WT1(+) | HAA | CR | dead |
| Patient.2# | Female | 48 | M5 | NA^2^ | NA | NA | CR | live |
| Patient.3# | Male | 80 | M2a | NA | NA | AZ (once) | NCR | dead |
| Patient.4# | Female | 49 | M2a | 46，XX | AML1-ET0(+)，WT1(+),FLT3-TKD(+) | HAAV | CR | live |
| Patient.5# | Female | 52 | M2a | 47,XX，+8/46,XX | WT1(+)FLT3-ITD(+),DNMT3A(+) | HAAV+Allo-HSCP | CR | live |
| Patient.6# | Male | 83 | M2a | 46,XY,t(8;21)(q22;q22)[9] | AML1-ETO (+)/WT1 (+) | VA | NCR | dead |
| Patient.7# | Male | 60 | M5 | 47，XY，+8，t（9；11）（p22；q23） | MLL-AF9(+),CSF3R(+) | VA+Allo-HSCP | CR | live |
| Patient.8# | Male | 65 | M2 | 46，XY，-7，+mar | WT1(+)，SETBP1(+)，NRAS(+)，PTPN11(+) | VA | NCR | dead |

Table S1. Clinical characteristics of primary AML patients
1. HAA: homoharringtonine+Cytarabine+aclacinomycin;

HAAV: homoharringtonine+Cytarabine+aclacinomycin+Venetoclax;

AZ: Cytarabine+Azacitidine;

VA:Venetoclax+Azacitidine;

1. NA:not available
